# Quality Evaluation of Wild and Cultivated *Schisandrae Chinensis* Fructus Based on Simultaneous Determination of Multiple Bioactive Constituents Combined with Multivariate Statistical Analysis

**DOI:** 10.3390/molecules24071335

**Published:** 2019-04-04

**Authors:** Shuyu Chen, Jingjing Shi, Lisi Zou, Xunhong Liu, Renmao Tang, Jimei Ma, Chengcheng Wang, Mengxia Tan, Jiali Chen

**Affiliations:** 1 College of Pharmacy, Nanjing University of Chinese Medicine, Nanjing 210023, China; 18305172513@163.com (S.C.); shijingjingquiet@163.com (J.S.); zlstcm@126.com (L.Z.); ccw199192@163.com (C.W.); 18816250751@163.com (M.T.); 18994986833@163.com (J.C.); 2 SZYY Group Pharmaceutical Limited, Taizhou 225500, China; ktang@vip.163.com (R.T.); mjm0607@hotmail.com (J.M.)

**Keywords:** *Schisandrae Chinensis* Fructus, wild, cultivated, multiple bioactive components, simultaneous quantitation

## Abstract

*Schisandrae Chinensis* Fructus, also called wuweizi in China, was a widely used folk medicine in China, Korea, and Russia. Due to the limited natural resources and huge demand of wuweizi, people tend to cultivate wuweizi to protect this species. However, the quality of wild and cultivated herbs of the same species may change. Little attention has been paid to comparing wild and cultivated wuweizi based on simultaneous determination of its active components, such as lignans and organic acids. An analytical method based on UFLC-QTRAP-MS/MS was used for the simultaneous determination of 15 components, including 11 lignans (schisandrin, gomisin D, gomisin J, schisandrol B, angeloylgomisin H, schizantherin B, schisanhenol, deoxyschizandrin, γ-schisandrin, schizandrin C, and schisantherin) and 4 organic acids (quinic acid, d(−)-tartaric acid, l-(−)-malic acid, and protocatechuic acid) in wuweizi under different ecological environments. Principal components analysis (PCA), partial least squares discrimination analysis (PLS-DA), independent sample t-test, and gray relational analysis (GRA) have been applied to classify and evaluate samples from different ecological environments according to the content of 15 components. The results showed that the differential compounds (i.e., quinic acid, l-(−)-malic acid, protocatechuic acid, schisandrol B) were significantly related to the classification of wild and cultivated wuweizi. GRA results demonstrated that the quality of cultivated wuweizi was not as good as wild wuweizi. The protocol not just provided a new method for the comprehensive evaluation and quality control of wild and cultivated wuweizi, but paved the way to differentiate them at the chemistry level.

## 1. Introduction

*Schisandrae Chinensis* Fructus (wuweizi) is the dried ripe fruit of the magnolia plant *Schisandra chinensis* (Turcz.) Baillon. Wuweizi was a folk medicine in China, Korea, and Russia, which was used as a sedative and tonic. With the ability to replenish vital energy and promote fluid production, benefitting the kidneys and tranquilizing the mind, wuweizi was used to treat seminal emission, excessive sweating, diarrhea, insomnia, fatigue, and neurasthenia in clinics [1,2]. Existing research studies have shown that lignans with a dibenzocyclooctadiene skeleton and organic acids are major types of phytochemicals in wuweizi [3,4]. It has been demonstrated that lignans have abundant bioactivities, including antihepatotoxic, antioxidant, antitumor, nervous system protection, and anticancer properties [5,6,7]. Organic acids have the beneficial pharmacological effects of arresting coughs and removing phlegm [2,8,9].

With the deeper understanding on the effectiveness of wuweizi, many industries, such as medicine, prescriptions, health products, and food and beverage, have a wide range of applications for this species. Various kinds of products, such as beverages, fermented wine, jam, tea, jelly, and dye, have aroused the demand for wuweizi. Due to excessive logging of unsustainable wild resources, the wild resources of wuweizi in northeast China have rapidly decreased and cannot meet the increasing demand of the markets. Therefore, planting cultivars has become a trend throughout northeast China. Unlike the wild ecological environment, the cultivated ecological environment is an artificial intervention. When plants face adversity, specialized metabolites accumulate significantly more than usual [10]. It is generally believed that most of the bioactive components isolated from herbs belong to specialised metabolites [11]. Varied ecological environments can lead to plants producing and accumulating various specialized metabolites, which cause uneven qualities of wuweizi. It is the amount or proportion among the medicinal constituents that mainly contribute to the quality difference for herbal medicine of the same species [12,13].

Nowadays, wuweizi is officially documented in Chinese, Japanese, European, Korean, and Russian pharmacopoeias, and other worldwide pharmacopoeial monographs [2]. Schisandrin is the quality indicator in the Chinese and United States pharmacopoeia. In addition, the content of lignans as the sum of the percentages of schisandrin, schisandrol B, deoxyschisandrin, and γ-schisandrin shall not be less than 0.95% by HPLC for quality control, as has been listed in the United States pharmacopeia. The control of several simple components may provide certain guidance, but for the multi-component and multi-target medicinal plants, there are still some limitations [14] because the pharmacological effect of wuweizi was usually a comprehensive effect of various kinds of compounds. For example, lignans and organic acids all have pharmacological effects, but they take different effects. However, most reports of quantitative analysis were focused on each particular class of compounds or certain active ingredients for the quality control of wuweizi [4,15,16,17,18]. The analysis of particular lignans alone was not sufficient for further study of the wuweizi quality control. Therefore, it is necessary to study the differences between wild and cultivated wuweizi and comprehensively evaluate the quality of them based on the simultaneous determination of lignans and organic acids.

The aim of this paper is to evaluate the quality of wild and cultivated wuweizi based on the simultaneous determination of multiple bioactive constituents combined with multivariate statistical analysis. A reliable method based on ultra-fast performance liquid chromatography coupled with triple quadrupole-linear ion trap mass spectrometry (UFLC-QTRAP-MS/MS) was used to simultaneously determine the content of 15 constituents, including 11 lignans and 4 organic acids in 12 batches of wuweizi samples from different ecosystems. Furthermore, principal component analysis (PCA) was introduced to get a good overview of the sample distribution. Partial least squares-discriminant analysis (PLS-DA) and t-tests were performed to show the difference of each compound between two types of wuweizi according to the contents of the tested constituents. Gray relational analysis (GRA) was carried out for the comprehensive evaluation. The protocol not just provided a new method for the comprehensive evaluation and quality control of wild and cultivated wuweizi, but allowed for differentiation of them at the chemistry level.

## 2. Results and Discussion

### 2.1. Optimization of Extraction Conditions

The orthogonal experiment was carried out to obtain a satisfactory extraction efficiency of major compounds in samples. Extraction solvent, material-solvent ratio, and ultrasonic time were optimized through 9 extraction experiments. The orthogonal table of levels and factors was shown in Table S1. The total peak area was calculated for evaluation of the orthogonal experiment, and the results were shown in Table S2. Finally, the optimum extraction conditions were achieved through ultrasonic extraction with a 40:1 ratio of methanol for 40 min at room temperature.

### 2.2. Optimization of UFLC Conditions

The peak area of schisandrin, theoretical plate number, resolution, and retention time were taken into consideration to investigate the different chromatographic columns, flow rates, column temperatures and mobile phases. Three kinds of columns, including an Agilent ZORBAX SB-C_18_ column (250 mm × 4.6 mm, 5 μm), a XBridge C_18_ (100 mm × 4.6 mm, 3.5 μm), and a Synergi^TM^ Hydro-RP 100Å (100 mm × 2.0 mm, 2.5 μm), were all compared to test samples. The results showed that the separation effect of three types of chromatographic columns were quite different, and the last one had the desirable resolution, peak shape, and retention time. Consequently, the column of Synergi^TM^ Hydro-RP 100Å (100 mm × 2.0 mm, 2.5 μm) was employed for this analysis. To achieve an efficient and rapid analysis, several UFLC parameters, including different kinds of mobile phases (methanol/water, acetonitrile/water, acetonitrile/0.1% aqueous formic acid, acetonitrile containing 0.1% formic acid solution/water, and acetonitrile containing 0.1% formic acid solution/0.1% aqueous formic acid), flow rates (0.3 mL/min, 0.35 mL/min and 0.4 mL/min), and column temperatures (30, 35, and 40 °C) were examined systematically. When acetonitrile aqueous solution was used for gradient elution, the shape of the peaks were better. When the flow rate was 0.40 mL/min, the resolution was better. The results of resolution under three column temperatures were all satisfactory, however, the column temperature of 40 °C had a higher theoretical plate number. Finally, it was determined that a gradient elution using water as eluent A and acetonitrile as eluent B at a flow rate of 0.4 mL/min under the column temperature of 40 °C resulted in the desired separation in a short analysis time. 

### 2.3. Optimization of MS Conditions

In order to develop a sensitive and accurate quantitative method, individual solutions of all standard compounds (about 100 ng/mL) were examined separately with infusion mode by a full-scan mass spectrometry method in both positive and negative modes. After trial and error inspection, we found that lignans show maximum sensitivity under the positive ion mode. However, organic acids display desirable results in the negative ion mode. ESI, the electrospray ionizationsource of MS, could obtain abundant fragment ions of compounds. MRM (multiple reaction monitoring) is suitable for the quantification of components as a promising technology for the sensitivity and robustness. MRM transition from the MS/MS spectrum was chosen when the most abundant fragment ions appeared. For example, compounds **1**, **2**, **3**, and **4** the showed most intense ion for [M − H]^−^. All the optimum values, including retention time (*t_R_*), mass data (*m*/*z*), precursor and product ions, fragmentor voltage (FV), and collision energy (CE) for each compound are summarized in Table 1. 

### 2.4. UFLC Method Validation

All method validations of quantification were performed using the UFLC-QTRAP-MS/MS technique. Each standard calibration curve was constructed by plotting the peak areas (Y) against the corresponding concentrations (X), which achieved appropriate determination coefficients (r^2^ > 0.9991), and the test range covered the concentrations of investigated compounds in samples. The limits of detection (LOD) and limits of quantification (LOQ) were in the ranges of 0.49–2.95 ng/mL and 1.38–12.65 ng/mL of 15 analytes, respectively. The relative standard deviation (RSD) values of intra-day and inter-day variations of 15 components ranged from 0.67% to 3.09% and 0.15% to 2.63%, respectively. The RSD of repeatability and stability tests of the 15 analytes were less than 5%, and the overall recoveries varied between 95.62% and 99.97%, with RSDs less than 4.12%, demonstrating that this method was validated for all kinds of analytes. The detailed results of each method validation were presented in Table 2 and Table S3. 

### 2.5. Quantification of Lignans and Organic Acids

**Figure 1:**
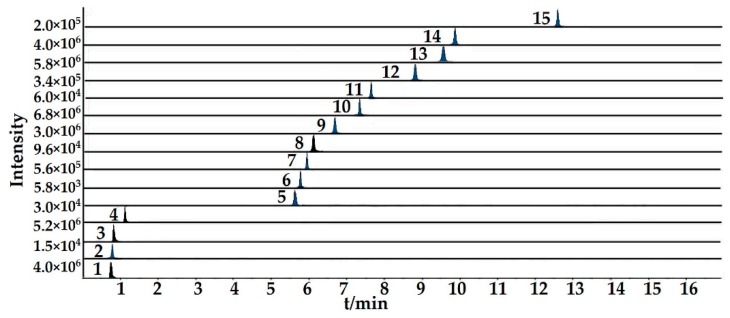
Representative extract ion chromatograms (XIC) of MRM chromatograms of 15 investigated compounds.

**Figure 2:**
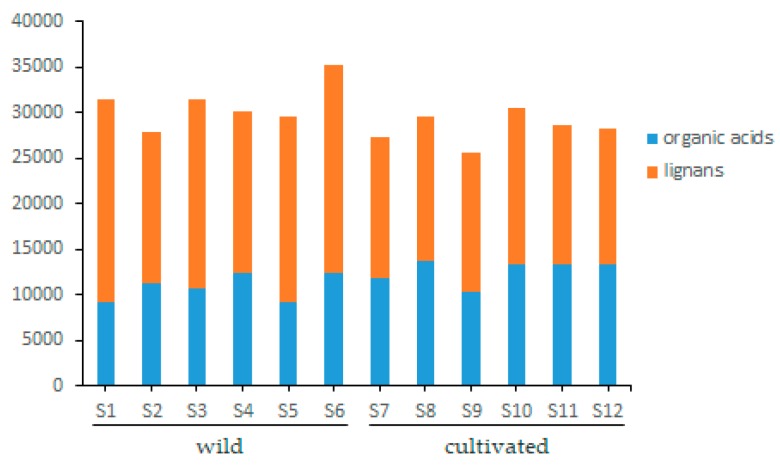
Histogram of the mean accumulative contents of different types of wuweizi from 12 batches.

Twelve batches of cultivated and wild wuweizi were collected from Heilongjiang, Jilin, and Liaoning and dealt with using the same processing method (sun drying). Sample information is listed in Table 3. The validated analytical method of UFLC-QTRAP-MS/MS was successfully applied to the simultaneous determination of 11 lignans and 4 organic acids in wuweizi. Each sample was determined three times and the quantitative results of 15 compounds are presented in Table 4. Total ion chromatograms of the representative wild and cultivated samples are shown in Figure S1. Typical MRM chromatograms are shown in Figure 1. The histogram (Figure 2) suggests lignans were found in higher concentrations in wild than cultivated-type. However, the content of organic acids was slightly higher in the cultivated-type. It was clearly shown that the total contents of 15 compounds varied from 25,598.77 μg/g to 35,179.73 μg/g. The total contents of each type of constituent was also calculated, and 11 lignans ranged from 14,960.30 μg/g to 22,853.62 μg/g, and in the following order: (highest) S1 (wild) > S6 (wild) > S3 (wild) > S5 (wild) > S4 (wild) > S10 (cultivated) > S2 (wild) > S8 (cultivated) > S7 (cultivated) > S11 (cultivated) > S9 (cultivated) > S12 (cultivated) (lowest). The four organic acids ranged from 9075.64 μg/g to 13,646.81 μg/g. By comparing the amounts, it was found that the compounds of wuweizi from different ecosystems were quite different. In this study, the contents of lignans were similar to previous studies [4,15,16,17,18]. However, the contents of organic acids were slightly lower than the reported, which may be related to the harvest period, processing, and origin [19].

### 2.6. PCA of the Samples

**Figure 3:**
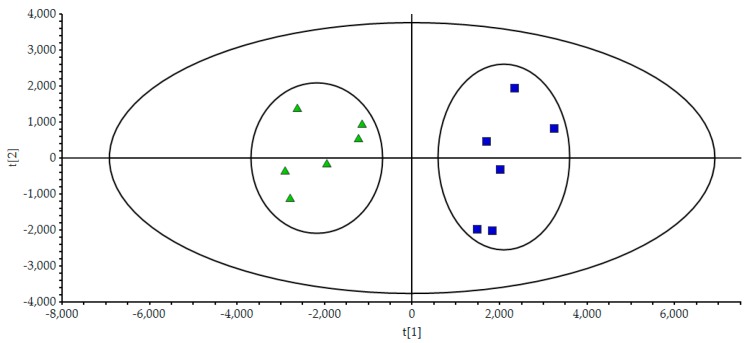
Score scatter plot of PCA processed data acquired from wild and cultivated wuweizi scanned by both positive and negative ion modes. Each of the green triangles represent a batch of wild wuweizi, while the blue squares represent a batch of cultivated ones.

To evaluate the differences of components in wuweizi of two types, unsupervised PCA was performed. 12 Samples were set as observations, while the contents of 15 compounds were set as variables. The score scatter plot and the loading plot were displayed in Figure 3. In Figure 3, all samples were separated into two relative clusters, i.e., wild and cultivated wuweizi. This classification indicated that the content and distribution of chemical constituents varied between different types of wuweizi. The first and second principal components described 59.4% and 17.6% of the variability in the original observations, respectively, and the first two principal components accounted for 77.0% of the total variance. 

### 2.7. PLS-DA of the Samples

**Figure 4:**
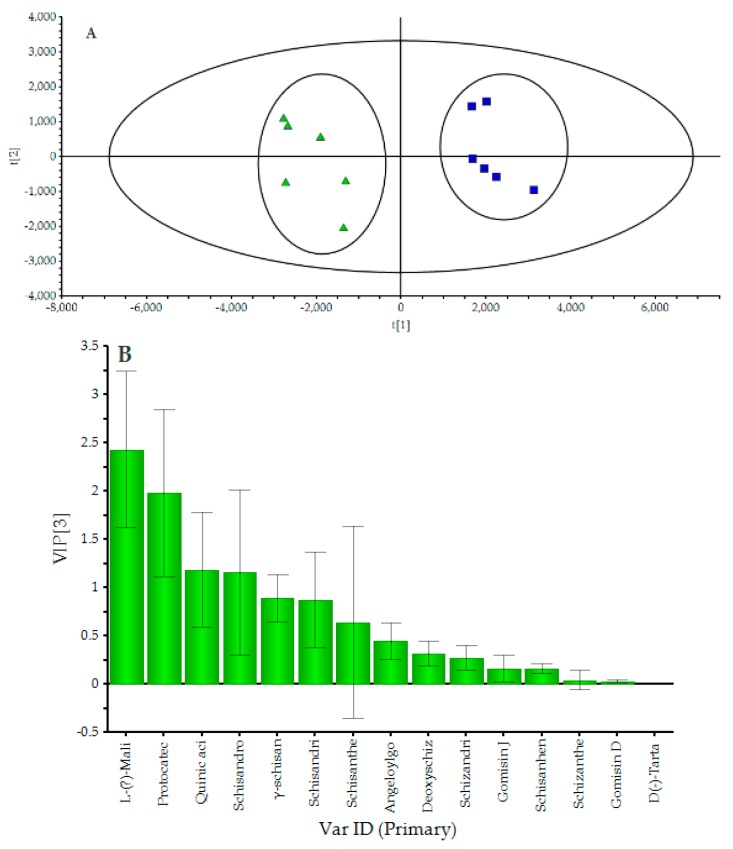
Score scatter plot (**A**) and VIP (**B**) by PLS-DA processed data obtained from wild and cultivated wuweizi scanned by positive and negative ion mode.

The selected initial data was further processed by PLS-DA in order to reveal differences in the chemical composition among wild and cultivated wuweizi. The results were shown in Figure 4. Model parameters were set as follows: confidence level was 95%, *R^2^Y* = 0.967, and *Q*^2^ = 0.924, and the parameters showed that the established PLS-DA model was effective. The PLS-DA score plot displayed that the two clusters representing the wild and cultivated groups were well separated, thereby indicating the remarkable differences between the two types.

The VIP (variable importance for the projection) plot summarizes the importance of the variables both to explain X and to correlate to Y. The VIP plot is arranged from high to low, and the VIP-values greater than 1 indicated important variables, and four potential biological markers of quinic acid, l-(−)-malic acid, protocatechuic acid, and schisandrol B have high contributions to classification.

### 2.8. T-test

**Figure 5:**
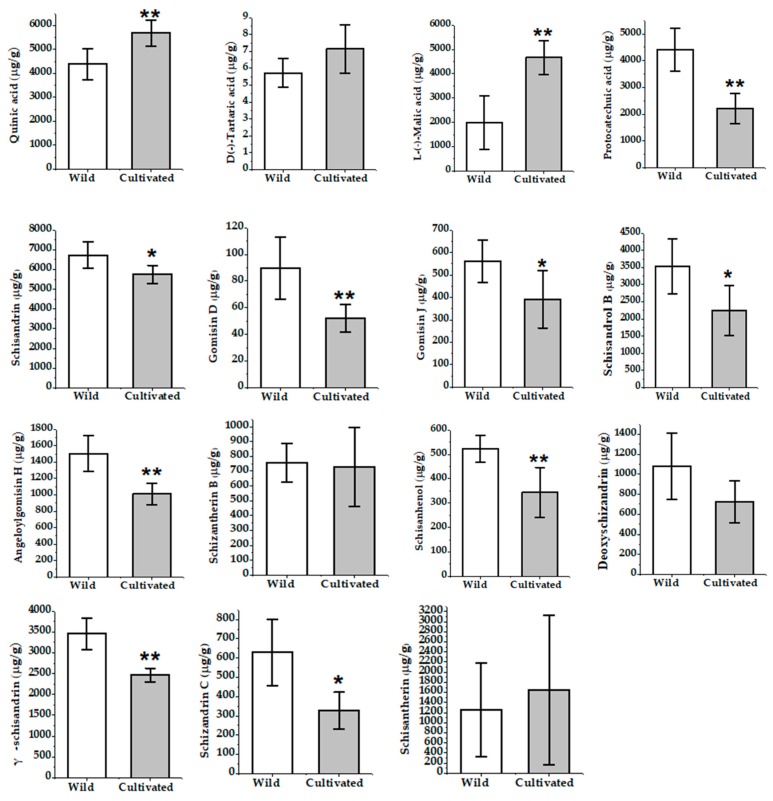
The contents of 15 compounds in wild and cultivated wuweizi (* *p* < 0.05; ** *p* < 0.01).

More than two-thirds of bioactive components quantified in this study showed a significant difference between two types of wuweizi, according to the T-test (Figure 5). Quantitation of major compounds, including protocatechuic acid, gomisin D, angeloylgomisin H, schisanhenol, and γ-schisandrin, showed strikingly higher levels (*p* < 0.01) in wild-type, while quinic acid and l-(−)-malic acid showed lower contents (*p* < 0.01) compared with cultivated-type. Secondly, quantitation of schisandrin, gomisin J, schisandrol B, and schizandrin C displayed highly contents (*p* < 0.05) in wild wuweizi than its cultivated-type. 

### 2.9. Gray Relational Analysis (GRA)

Because the contents of the 15 target components of lignans and organic acids in samples were different, it is difficult to judge the quality of samples intuitively. Therefore, gray relational analysis was carried out for comprehensive evaluation. It could be seen that the quality of wild wuweizi was better according to the results shown in Table S4. The quality sequencing of the samples was in the following order: S1 (wild) *>* S6 (wild) *>* S3 (wild) *>* S4 (wild) *>* S5 (wild) *>* S2 (wild) *>* S10 (cultivated) *>* S12 (cultivated) *>* S8 (cultivated) *>* S11 (cultivated) *>* S7 (cultivated) *>* S9 (cultivated).

## 3. Materials and Methods

### 3.1. Plant Materials

Twelve batches of cultivated and wild wuweizi dealing with the same processing method were studied in this research. The botanical origin of materials was authenticated by Prof. Xunhong Liu of the Nanjing University of Chinese Medicine. Voucher specimens were deposited at Herbarium in Nanjing University of Chinese Medicine. These samples were collected from Heilongjiang, Jilin, and Liaoning at around August 2017, dealing with sun drying for about 20 days. All batches of wuweizi were ripe when they were collected. Wild wuweizi grew under forests, valleys, and besides streams, places which were shady and moist. Cultivated wuweizi grew in arable land, which had enough sunshine and wind, or low-lying and rainy land. Detailed information is shown in Table 3.

### 3.2. Chemicals and Reagents

**Figure 6:**
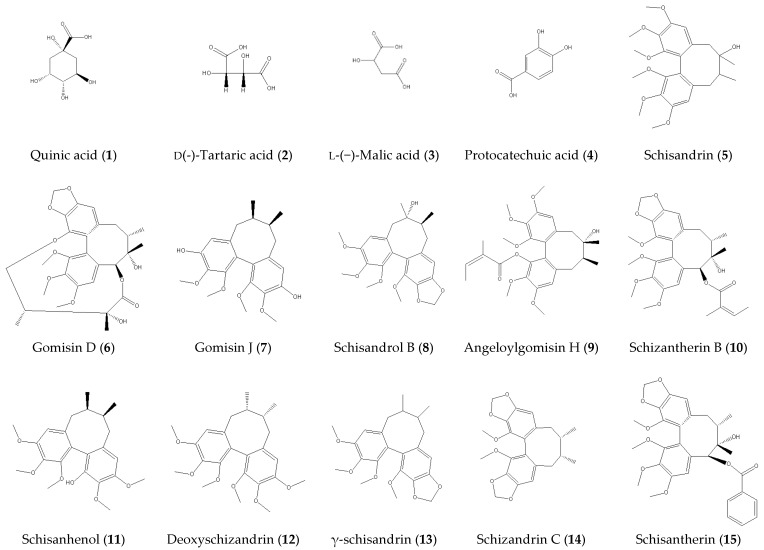
Chemical structures of 15 reference substances.

The reference compounds of l-(−)-malic acid (**3**), deoxyschizandrin (**12**), γ-schisandrin (**13**), schisandrin (**5**), schisantherin (**15**) were purchased from National Institutes for Food and Drug Control (Beijing, China). Quinic acid (**1**), d(-)-Tartaric acid (**2**), gomisin D (**6**), gomisin J (**7**), schizandrin C (**14**), schisandrol B (**8**), angeloylgomisin H (**9**), schizantherin B (**10**), and schisanhenol (**11**) were purchased from Nanjing Liangwei biotechnology Co., Ltd. (Nanjing, China). Protocatechuic acid (**4**) was purchased from Nanjing Spring and Autumn Biological Engineering Co., Ltd., China. The purity of all compounds by HPLC analysis was greater than 98%. The structures of the 15 reference compounds are shown in Figure 6. Formic acid of MS grade, acetonitrile, and methanol of HPLC grade were purchased from Merck (Darmstadt, Germany). Ultrapure water was prepared using a Milli-Q water purification system (Millipore, Bedford, MA, USA). 

### 3.3. Preparation of Standard Solutions

A mixed standard stock solution containing 15 reference standards was prepared in methanol and their concentrations were as follows: **1**, 26.60 μg/mL; **2**, 0.10 μg/mL; **3**, 29.52 μg/mL; **4**, 12.77 μg/mL; **5**, 9.31 μg/mL; **6**, 1.01 μg/mL; **7**, 0.93 μg/mL; **8**, 5.35 μg/mL; **9**, 2.26 μg/mL; **10**, 2.21 μg/mL; **11**, 1.20 μg/mL; **12**, 2.40 μg/mL; **13**, 4.60 μg/mL; **14**, 1.47 μg/mL; **15**, 7.17 μg/mL. This standard stock solution was then diluted with methanol to a series of appropriate concentrations to generate the calibration curves. The solutions were stored at 4 °C for a day prior to LC-MS analysis.

### 3.4. Preparation of Sample Solutions

The dried fruits were pulverized into powders and screened through the 50-mesh sieve. Each sample (0.5 g) was accurately weighed and extracted by ultrasonication (500 W, 40 kHz) in 20 mL methanol for 40 min. After cooling down at room temperature, methanol was added to compensate for the weight lost during extraction. After centrifugation (12,000 rpm, 10 min) and filtering (0.22 μm membrane filter), the supernatants were stored in a sample plate at 4 °C prior to LC-MS analysis.

### 3.5. Chromatographic and Mass Spectrometric Conditions

The mass spectrometry detection was performed using an API5500 triple quadrupole mass (AB SCIEX, Framingham, MA, USA). The MS was equipped with an electrospray ionization (ESI) source operating in MRM and under both positive and negative ion modes. The MS parameters were set as follows: gas temperature 550 °C; pressures of nebulizer of MS, 5500 V (positive) and −4500 V (negative); GSI flow 65 L/min; CUR flow 30 L/min and all MS data were acquired and analyzed using the Analyst 1.5.2 software (AB SCIEX, Framingham, MA, USA). The cone voltage and collision energy parameter of each compound were individually optimized.

The chromatographic analysis was performed on a Shimadzu SIL-20A XR system (Shimadzu, Kyoto, Japan), consisting of a binary solvent delivery system and an automatic sampler. A Synergi^TM^ Hydro-RP 100Å column (100 mm × 2.0 mm, 2.5 μm, (Phenomenex, Los Angeles, CA, USA) was used for eluting samples. The mobile phase was composed of water (A) and acetonitrile (B) using a gradient elution of 30%–52% B at 0–4 min, 52%–75% B at 4–8 min, 75%–90% B at 8–11 min, 90%–30% B at 11–15 min, 30% B at 15–17.10 min. The column temperature was 40 °C, the flow rate kept at 0.4 mL/min, and the sample injection volume was 1 μL.

### 3.6. Validation of the Method

#### 3.6.1. Linearity, LOD, and LOQ

The linearity of the calibration curves was obtained by plotting the peak areas (Y) against the corresponding concentrations (X) of each analyte. The lowest concentration of standard solution for calibration use was diluted with methanol to a series of appropriate concentrations. The LODs and LOQs of 15 analytes were determined using a series of diluted standard solutions until the signal-to-noise (S/N) ratios were about 3 and 10, respectively. 

#### 3.6.2. Precision, Repeatability, Stability, Accuracy

The analysis method developed in this study was validated for precision (the intra- and inter-day), repeatability, stability, and accuracy. The intra-day and inter-day variability tests were determined by measuring the mixed standard solutions in six replicates in a day and once a day during three consecutive days, respectively. To evaluate the repeatability, six different analytical sample solutions prepared from the same sample (sample 1) were parallel processed and analyzed. To confirm the stability, the sample solution mentioned above was stored at room temperature and analyzed at 0, 2, 4, 8, 12, and 24 h, respectively. All the variations were expressed in RSD. A recovery test was used to evaluate the accuracy of this method. A certain amount of the 15 standards with low (80%), medium (100%), and high (120%) levels were added into a known amount of samples (0.25 g), and then extracted and analyzed with the same procedures. To be specific, a recovery test was conducted by standard protocol and calculated by the formula: (%) = (found amount– original amount in sample)/spiked amount × 100%. 

### 3.7. Multivarite Statistical Analysis

Multivariate statistical analysis was performed using the Simca-P 13.0 software (for Windows, Umetrics AB, Umeå, Sweden) by PCA and PLS-DA. PCA and PLS-DA were used to evaluate the variations of the two types of wuweizi according to the contents of the 15 components. PCA is an unsupervised pattern recognition method used for analyzing, classifying, and reducing the dimensionality of numerical datasets in a multivariate problem [20,21], and it has been widely used for the quality control of herbal medicines [22,23,24]. PLS-DA is good for highlighting the differences between two groups. It is possible to identify and select the important markers in samples via multivariate analysis of LC/MS data, even at low concentration levels [25]. Data of the contents of 15 compounds in wild and cultivated samples were listed. When the contents of investigated components were below the quantitation limit or not detected in the samples, the values of such elements were considered to be 0. All experimental data were statistically analyzed by independent sample t-test (SPSS 16.0 for Windows, IBM, Armonk, NY, USA). The columns were charted by Origin pro 8 (Origin Lab, Northampton, MA, USA), showing the difference of each compound between two types of wuweizi. GRA provides a reliable guarantee for the quality evaluation of traditional Chinese medicine on the basis of the contents of the 15 index constituents [26].

## 4. Conclusions

An analytical method based on UFLC-QTRAP-MS/MS was used for the simultaneous determination of 15 components, including 11 lignans and 4 organic acids, in wuweizi under different ecosystems (wild and cultivated). Multivariate statistical analyses, such as PCA, PLS-DA, independent sample t-test, and GRA, have been successfully applied to comprehensively analyze and evaluate the wuweizi under different ecosystems according to the contents of the 15 components. The data of content determination showed that lignans had higher contents in wild-type, while cultivated-type contained more organic acids. This phenomenon is probably ascribed to environmental stress (such as water, soil strength, and nutrient status), which can have a great influence on the accumulation of the active compounds of medicinal plants. PCA and PLS-DA results showed that there are great difference between wild and cultivated wuweizi, and 4 different compounds (protocatechuic acid, quinic acid and l-(−)-malic acid, and schisandrol B) were significantly related to sample classification. It could be seen that the quality of the samples collected from the wild environment was better according to the GRA results. Wild wuweizi usually suffered more stress than cultivated-type and produced more specialized metabolites, even though the quality indicator of the two types all conformed to the requirements of Chinese pharmacopoeia. Findings from this research may provide a new method for the comprehensive evaluation and quality control of wuweizi from different ecosystems. It may also provide a basis for differentiating wild and cultivated wuweizi at the chemistry level.

## Figures and Tables

**Table 1:** Precursor/product ion pairs and parameters for MRM of the target compounds.

No	Coumpounds	*t_R_* (min)	Mass Data (*m*/*z*)	Precursor Ion	Product Ion	FV (V)	CE (eV)
**1**	Quinic acid	0.79	191.1[M − H]−	191.1	85.02	−120	−26
**2**	d(−)-Tartaric acid	0.82	149.0[M − H]−	149.0	87	−55	−16
**3**	l-(−)-Malic acid	0.84	133.0 [M − H]−	133.0	114.9	−80	−14
**4**	Protocatechuic acid	1.11	153.0 [M − H]−	153.0	106	−85	−16
**5**	Schisandrin	5.65	433.3[M + H]^+^	433.3	415.34	146	13
**6**	Gomisin D	5.75	553.3[M + Na]^+^	553.3	507.32	21	35
**7**	Gomisin J	5.92	389.3[M + H]^+^	389.3	287.1	156	27
**8**	Schisandrol B	6.11	417.3[M + H]^+^	417.3	399.2	131	15
**9**	Angeloylgomisin H	6.67	501.3[M + H]^+^	501.3	401.2	146	11
**10**	Schizantherin B	7.34	515.3[M + H]^+^	515.3	415.2	56	11
**11**	Schisanhenol	7.63	403.2[M + H]^+^	403.2	340.2	1	27
**12**	Deoxyschizandrin	8.83	417.3[M + H]^+^	417.3	316.18	241	31
**13**	γ-schisandrin	9.50	401.3[M + H]^+^	401.3	300.15	231	31
**14**	Schizandrin C	9.85	385.2[M +H]^+^	385.2	285.16	201	29
**15**	Schisantherin	11.96	537.4[M + H]^+^	537.4	282.3	56	15

**Table 2:** Regression equation, limits of detection, limits of quantification, precision, repeatability, stability, and recovery of 30 investigated compounds.

No.	Compounds	Regression Equation	r^2^	Liner Range (ng/mL)	LODs (ng/mL)	LOQs (ng/mL)	Precision (RSD, %)		Repeatability (RSD, %) (*n* = 6)	Stability (RSD, %) (*n* = 6)	Recovery (%) (*n* = 3)					
							Intra-Day (*n* = 6)	Inter-Day (*n* = 3)			Low		Medium		High	
											Mean	RSD	Mean	RSD	Mean	RSD
**1**	Quinic acid	Y = 1330X + 76100	1.0000	133 – 13300	2.66	6.57	2.40	2.63	3.5	1.1	99.55	0.22	99.65	0.36	99.83	0.05
**2**	d(−)-Tartaric acid	Y = 1240X + 1190	0.9995	3.75 – 24.9	0.49	1.62	3.09	1.60	2.1	2.3	95.62	2.1	98.07	0.57	97.34	0.74
**3**	l-(−)-Malic acid	Y = 2040X + 149000	0.9991	148 – 29500	2.95	12.42	1.79	1.45	3.2	1.2	99.92	0.06	99.93	0.03	99.96	0.06
**4**	Protocatechuic acid	Y = 162X + 3100	0.9997	255 – 12800	1.96	9.03	2.31	1.27	2.5	3.3	99.98	0.01	99.96	0.02	99.93	0.06
**5**	Schisandrin	Y = 21.3X + 1550	0.9991	11.6 – 9320	1.36	7.84	3.06	1.86	0.7	3.1	99.36	0.3	99.8	0.1	99.63	0.06
**6**	Gomisin D	Y = 3770X + 34900	0.9992	10.1 – 506	0.84	6.61	1.11	0.81	1.5	1.2	96.63	2.36	96.96	1.87	96.33	4.12
**7**	Gomisin J	Y = 739X + 7100	0.9993	23.3 – 931	1.53	6.07	2.03	1.26	3.0	3.0	98.63	0.62	98.59	0.54	97.7	0.45
**8**	Schisandrol B	Y = 4140X + 260000	0.9997	107 – 5350	1.92	8.15	2.56	0.31	2.4	0.8	99.05	0.38	98.85	0.18	99.13	0.7
**9**	Angeloylgomisin H	Y = 25800X + 623000	0.9991	5.65 – 2260	1.36	5.38	2.45	0.15	1.6	3.1	99.84	0.22	99.24	0.74	99.68	0.32
**10**	Schizantherin B	Y = 240X + 3640	0.9998	55.2 – 2210	2.29	12.65	1.00	0.54	1.3	2.3	99.02	0.34	98.98	0.73	99.33	0.69
**11**	Schisanhenol	Y = 2900X + 10800	0.9999	19.9 – 1194	1.99	5.82	2.59	1.58	4.9	1.5	98.19	1.12	98.63	0.88	96.92	3.3
**12**	Deoxyschizandrin	Y = 26600X + 217000	1.0000	24 – 2400	2.91	11.21	0.87	1.10	1.6	1.3	97.83	0.74	98.86	0.34	99.42	0.02
**13**	γ-schisandrin	Y = 15700X + 214000	1.0000	15.2 – 4560	1.79	6.92	1.91	0.43	0.5	1.1	99.63	0.3	99.75	0.09	99.69	0.13
**14**	Schizandrin C	Y = 6410X + 24300	0.9991	29.3 – 1470	0.52	1.38	2.25	0.66	3.8	2.5	98.57	0.41	97.64	0.61	98.24	0.61
**15**	Schisantherin	Y = 25X +1860	0.9998	12 – 7200	1.59	5.62	0.67	1.61	2.2	1.0	99.89	0.04	99.89	0.04	99.97	0.02

**Table 3:** Detailed information of samples.

Sample No.	Habitats	GPS Records	Harvesting Time	Processing Method
S1	Mulan, Heilongjiang	45°56′54″ N, 128°02′14″ E	10 August 2017	sun drying
S2	Jingyu, Jilin	42°23′11″ N, 126°48′28″ E	14 August 2017	sun drying
S3	Jingyu, Jilin	42°23′11″ N, 126°48′28″ E	17 August 2017	sun drying
S4	Xinbin, Liaoning	41°43′53″ N, 125°02′01″ E	14 August 2017	sun drying
S5	Hengren, Liaoning	41°15′13″ N, 125°22′15″ E	10 August 2017	sun drying
S6	Baoqing, Heilongjiang	46°19′29″ N, 132°11′22″ E	20 August 2017	sun drying
S7	Jingyu, Jilin	42°23′11″ N, 126°48′28″ E	16 August 2017	sun drying
S8	Jingyu, Jilin	42°23′11″ N, 126°48′28″ E	17 August 2017	sun drying
S9	Jingyu, Jilin	42°23′11″ N, 126°48′28″ E	20 August 2017	sun drying
S10	Fengcheng, Liaoning	41°48′19″ N, 123°27′47″ E	10 August 2017	sun drying
S11	Shuangyang, Jilin	43°31′22″ N, 125°39′31″ E	10 August 2017	sun drying
S12	Heihe, Heilongjiang	50°14′37″ N, 127°31′16″ E	16 August 2017	sun drying

**Table 4:** Contents of 15 compounds in wuweizi (μg/g, mean ± SD, *n* = 3).

Analyte	Wild						Cultivated					
	S1 ^a^	S2 ^a^	S3 ^a^	S4 ^a^	S5 ^a^	S6 ^a^	S7 ^a^	S8 ^a^	S9 ^a^	S10 ^a^	S11 ^a^	S12 ^a^
**1** ^b^	5093.16 ± 30.08	4213.46 ± 7.52	4283.63 ± 18.92	5155.81 ± 4.34	3461.58 ± 52.63	4070.60 ± 22.56	5108.20 ± 15.04	5243.53 ± 30.08	5228.50 ± 22.56	6220.98 ± 15.04	6175.86 ± 22.56	6115.71 ± 0.00
**2** ^b^	5.27 ± 0.04	6.23 ± 0.01	6.45 ± 0.04	6.80 ± 0.03	5.05 ± 0.01	4.73 ± 0.01	8.15 ± 0.48	8.31 ± 0.32	4.60 ± 0.06	7.06 ± 0.01	6.69 ± 0.03	8.31 ± 0.32
**3** ^b^	657.03 ± 0.57	2050.65 ± 1.98	2687.42 ± 1.13	1564.71 ± 0.85	1245.42 ± 0.28	3781.21 ± 2.26	4196.57 ± 8.49	5413.89 ± 5.66	3585.78 ± 5.09	5379.58 ± 19.81	4673.53 ± 2.55	4884.48 ± 11.32
**4** ^b^	3382.10 ± 24.69	5061.52 ± 0.71	3680.04 ± 2.85	5562.96 ± 3.21	4363.58 ± 0.00	4469.14 ± 1.07	2470.58 ± 7.13	2981.07 ± 0.36	1426.13 ± 1.43	1716.67 ± 2.14	2408.23 ± 2.49	2290.53 ± 1.78
**5** ^b^	6171.36 ± 46.95	5847.42 ± 72.88	7110.33 ± 37.56	7287.17 ± 9.77	6457.75 ± 4.69	7482.79 ± 5.42	5847.42 ± 54.14	6451.49 ± 2.71	5093.11 ± 49.31	5988.26 ± 101.02	5847.42 ± 16.93	5510.95 ± 42.34
**6** ^b^	107.63 ± 0.31	64.04 ± 0.15	61.48 ± 1.6	94.81 ± 0.67	93.75 ± 1.62	119.30 ± 0.93	37.16 ± 0.46	63.69 ± 0.80	42.29 ± 0.77	54.58 ± 0.31	60.86 ± 0.41	54.49 ± 0.55
**7** ^b^	662.92 ± 2.34	601.58 ± 0.78	562.34 ± 6.1	389.13 ± 2.07	539.33 ± 4.35	617.82 ± 2.82	312.90 ± 3.91	309.29 ± 3.13	260.13 ± 1.56	478.89 ± 4.06	600.68 ± 3.58	382.81 ± 1.35
**8** ^b^	4800.32 ± 27.89	2537.84 ± 13.95	3528.18 ± 13.95	2859.90 ± 48.31	3560.39 ± 53.14	3850.24 ± 48.31	1512.88 ± 1.39	1762.48 ± 6.08	1802.74 ± 2.79	2029.79 ± 3.69	3278.58 ± 27.89	3061.19 ± 60.79
**9** ^b^	1814.35 ± 5.92	1216.16 ± 38.76	1518.49 ± 3.88	1344.07 ± 11.63	1446.14 ± 8.07	1690.97 ± 22.35	934.51 ± 13.61	916.42 ± 4.48	878.95 ± 15.50	1014.61 ± 7.75	1182.83 ± 2.05	1147.69 ± 2.24
**10** ^b^	840.39 ± 2.41	547.33 ± 4.17	877.89 ± 10.49	684.83 ± 15.02	870.94 ± 18.79	714.00 ± 12.50	714.00 ± 12.50	557.06 ± 6.36	458.44 ± 18.79	726.50 ± 8.33	690.39 ± 12.73	1205.67 ± 36.32
**11** ^b^	626.16 ± 5.27	528.46 ± 10.53	523.86 ± 6.90	485.93 ± 10.34	468.69 ± 3.45	508.92 ± 12.11	457.20 ± 8.68	481.33 ± 1.99	268.92 ± 0.80	341.1 ± 0.00	263.63 ± 0.53	245.59 ± 0.34
**12** ^b^	1749.99 ± 2.17	978.71 ± 4.34	974.32 ± 2.56	965.35 ± 3.76	849.74 ± 10.85	971.36 ± 41.41	916.18 ± 5.74	1028.87 ± 1.30	602.78 ± 4.24	729.21 ± 3.39	538.68 ± 4.7	521.74 ± 3.57
**13** ^b^	4046.88 ± 3.18	3149.85 ± 3.68	3165.77 ± 1.84	3158.56 ± 0.37	3504.42 ± 9.73	3774.06 ± 16.03	2635.63 ± 0.74	2647.94 ± 5.15	2208.87 ± 5.74	2495.71 ± 6.05	2489.55 ± 6.37	2374.27 ± 5.44
**14** ^b^	787.58 ± 1.00	333.70 ± 0.90	689.40 ± 2.38	516.23 ± 3.25	704.79 ± 2.05	737.24 ± 5.40	249.41 ± 0.95	281.70 ± 1.56	247.38 ± 0.00	277.80 ± 0.78	437.55 ± 1.58	455.91 ± 5.01
**15** ^b^	705.60 ± 12.00	708.27 ± 2.31	1692.27 ± 2.31	-	2046.93 ± 8.33	2386.93 ± 6.11	1889.60 ± 4.00	1421.60 ± 0.00	3490.93 ± 4.62	3093.60 ± 6.93	19.87 ± 1.01	-
Total	31,450.74 ± 55.65	27,844.58 ± 40.31	31,361.47 ± 100.10	30,076.44 ± 28.88	29,619 ± 105.38	35,179.73 ± 61.85	27,289.59 ± 94.08	29,568.49 ± 19.79	25,598.77 ± 42.20	30,553.57 ± 169.43	28,674.25 ± 21.71	28,259.65 ± 12.66

^a^ The sample number is same as in Table 3; ^b^ the analyte number is the same as in Table 2.

## Supplementary Materials

The following are available online. Table S1: Levels and factors of orthogonal table. Table S2: Result of L_9_(3)^3^ orthogonal experiment. Table S3: Recoveries and relative standard deviations (RSD) of fifteen components (%, *n* = 3). Table S4: Quality sequencing of the samples. Figure S1: Total ion chromatogram of the representative wild and cultivated samples.
