# Supplementary material for: Quality Evaluation of Wild and Cultivated Schisandrae Chinensis Fructus Based on Simultaneous Determination of Multiple Bioactive Constituents Combined with Multivariate Statistical Analysis
Source: Molecules. 2019 Apr 4;24(7):1335. doi: 10.3390/molecules24071335 (PMC6479832; doi:10.3390/molecules24071335)
Supplement: Supplementary file 1 [file molecules-24-01335-s001.pdf]

Supplementary materials

Quality Evaluation of Wild and Cultivated Schisandrae Chinensis Fructus Based on Simultaneous Determination of Multiple Bioactive Constituents Combined with Multivariate Statistical Analysis

Shuyu Chen [1], Jingjing Shi [1], Lisi Zou [1], Xunhong Liu [1]*, Renmao Tang [2], Jimei Ma [2], Chengcheng Wang [1], Mengxia Tan [1] and Jiali Chen [1]

1 College of Pharmacy, Nanjing University of Chinese Medicine, Nanjing 210023, China; 18305172513@163.com (S.C.); shijingjingquiet@163.com (J.S.); zlstcm@126.com (L.Z.); ccw199192@163.com (C.W.); 18816250751@163.com (M.T.); 18994986833@163.com (J.C.);

2 SZYY Group Pharmaceutical Limited, Taizhou 225500, China; ktang@vip.163.com (R.T.); mjm0607@hotmail.com (J.M.);

* Correspondence: liuxunh1959@163.com; Tel./Fax: +86-25-8581-1524

Table 1. Levels and factors of orthogonal table.

Level	Factor		
	Methanol concentration (A, %)	Sample-solvent ratio (B)	Ultrasonic time (C, min)
1	50	1:20	20
2	70	1:40	40
3	100	1:60	60

Table S2. Result of $L_9\,(3)^3$ orthogonal experiment

Group	Factor			Total peak area
	A	B	C	
1	A_1	B_1	C_1	118.2
2	A_1	B_2	C_2	120.2
3	A_1	B_3	C_3	109.6
4	A_2	B_1	C_3	115.6
5	A_2	B_2	C_1	116.6
6	A_2	B_3	C_2	104.8
7	A_3	B_1	C_2	123.1
8	A_3	B_2	C_3	120.5
9	A_3	B_3	C_1	119.5
K_1	116.000	118.967	114.500	
K_2	112.333	119.100	118.433	
K_3	121.033	111.300	116.433	
R	8.700	7.800	3.933	
Optimal level	A_3	B_2	C_2	
Important order		A>B>C		

Table S3. Recoveries and relative standard deviations (RSD) of 15 components (%, n=3)

Compounds	Content in sample (μg)	Standard added (μg)	Measured	Recovery	Average recovery ± SD
Quinic acid	1265.222	1012.700	2273	99.55	99.67±0.25
	1265.391	1265.400	2526	99.65	
	1265.222	1519.050	2273	99.83	
D(-)-Tartaric acid	1.939	0.998	2.89	95.62	97.01±1.6
	1.939	1.996	3.90	98.07	
	1.939	2.994	4.85	97.34	
(S)-malic acid	691.255	553.268	1244	99.92	99.94±0.05
	691.348	691.585	1382	99.93	
	691.255	829.902	1520	99.96	
Protocatechuic acid	1986.424	1589.742	3576	99.98	99.96±0.04
	1986.688	1987.233	3973	99.96	
	1986.424	2384.613	4369	99.93	
Schisandrin	1594.629	1276.520	2863	99.36	99.96±0.25

	1594.841	1595.650	3187	99.80	
	1594.629	1914.780	3502	99.63	
Gomisin D	32.442	25.760	57	96.63	96.64±2.57
	32.446	32.200	64	96.96	
	32.446	38.640	70	96.33	
Gomisin J	111.050	88.830	199	98.63	98.31±0.66
	111.065	110.826	220	98.59	
	111.080	133.668	242	97.70	
Schisandrol B	631.363	505.440	1132	99.05	99.01±0.43
	631.447	631.800	1256	98.85	
	631.447	758.160	1383	99.13	
Angeloylgomisin H	272.010	218.004	490	99.84	99.59±0.5
	272.046	272.014	542	99.24	
	272.046	327.006	598	99.68	
Schizantherin B	217.608	173.760	390	99.02	99.11±0.55
	217.637	217.920	433	98.98	
	217.637	261.120	477	99.33	
Schisanhenol	68.876	54.780	123	98.19	97.92±1.95
	68.885	68.724	137	98.63	
	68.876	82.668	149	96.92	
Deoxyschizandrin	166.450	133.440	297	97.83	98.71±0.82
	166.472	166.080	331	98.86	
	166.472	199.680	365	99.42	
γ-schisandrin	756.139	604.404	1358	99.63	99.69±0.18
	756.239	755.994	1510	99.75	
	756.239	907.584	1661	99.69	
Schizandrin C	162.458	130.074	291	98.57	98.15±0.63
	162.479	162.348	321	97.64	
	162.479	194.622	354	98.24	
Schisantherin	743.661	595.149	1338	99.89	99.92±0.05
	743.760	743.936	1487	99.89	
	743.661	892.723	1636	99.97	

Table S4. Quality sequencing of the samples

**Items**	**S1** [a]	**S2** [a]	**S3** [a]	**S4** [a]	**S5** [a]	**S6** [a]	**S7** [a]	**S8** [a]	**S9** [a]	**S10** [a]	**S11** [a]	**S12** [a]
Relative gray correlative degree	0.6041	0.4562	0.5335	0.5033	0.4988	0.5865	0.4109	0.4525	0.3279	0.4559	0.4418	0.4525
Quality ranking	1	6	3	4	5	2	11	9	12	7	10	8

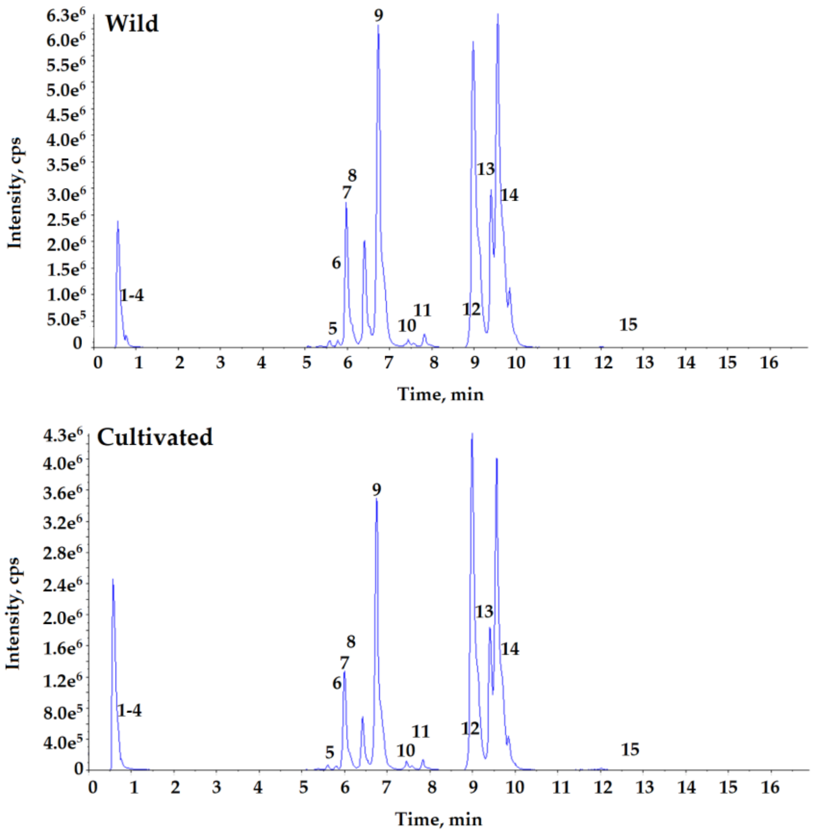

Figure S1. Total ion chromatogram of the representative wild and cultivated samples
